# Increasing Temperature Facilitates Polyp Spreading and Medusa Appearance of the Invasive Hydrozoan *Craspedacusta sowerbii*

**DOI:** 10.3390/biology11081100

**Published:** 2022-07-23

**Authors:** Guillaume Marchessaux, Florian Lüskow, Mickaël Bejean, Evgeny A. Pakhomov

**Affiliations:** 1 Department of Earth and Marine Science, University of Palermo, Viale delle Scienze, 90128 Palermo, Italy; 2 Department of Earth, Ocean and Atmospheric Sciences, University of British Columbia, 2039-2207 Main Mall, Vancouver, BC V6T 1Z4, Canada; flueskow@eoas.ubc.ca (F.L.); evgeny.pakhomov@ubc.ca (E.A.P.); 3 Institute for the Oceans and Fisheries, University of British Columbia, 2202 Main Mall, Vancouver, BC V6T 1Z4, Canada; 4 Muséum de Besançon, 99 Rue Des Fusillés, La Citadelle, 25000 Besançon, France; mickael.bejean@citadelle.besancon.fr; 5 Hakai Institute, Heriot Bay, BC V0P 1H0, Canada

**Keywords:** climate change, invasive hydrozoan, species distribution modeling, thermal tolerance

## Abstract

**Simple Summary:**

The jellyfish *Craspedacusta sowerbii* is one of the most widespread freshwater invasive species. The aim of this study is to analyze polyp and medusa responses to different temperatures using experimental studies on polyp colony growth and medusae to develop a model capable of predicting the Thermal Habitat Suitability (THS) for the polyp and medusa stages. Temperature had a significant effect on the total number of polyps and colonies over time and on the structure of polyp colonies. Polyp and colony numbers were greater at high temperature while colonies were composed of more polyps per colony at 19 °C compared to 29 °C. The thermal tolerance of the medusa stage showed that temperature will favor the expansion of the species in the future to higher latitudes.

**Abstract:**

The freshwater jellyfish *Craspedacusta sowerbii* is among the most widespread invasive species, observed across a wide temperature range. The aim of this study is to analyze the polyp and medusa stages response to different temperatures by using (i) an experimental study on the polyp colony growth at 19 and 29 °C, and (ii) prediction of the Thermal Habitat Suitability (THS) based on the thermal tolerance of the medusa stage. The total number of polyps and colonies was greater at high temperature. At 19 °C, colonies with 1 to 5 polyps were present, with colonies of 1 to 3 polyps numerically dominating. At 29 °C, colonies were 80% composed of 1- to 2-polyps. Based on the published medusa pulsation rhythm data, a Thermal Performance Curve (TPC) regression was performed and used to monthly predict the THS for current and future (2050 and 2100) scenarios. The southern hemisphere offered optimal conditions (THS > 0.6) year-round. In the northern hemisphere, the optimum period was predicted to be between June and September. The future THS were considerably larger than at present with an increase in optimal THS at higher latitudes (up to 60° N). The combination of experimental and modeling approaches allows to identify the optimal thermal conditions of the polyp and medusa stages and to predict their invasive capacities.

## 1. Introduction

The global rise in temperatures affects organismal performance, population dynamics, and the ecosystem structure [1,2], facilitating an expansion of the organisms’ distribution towards the poles [3,4,5]. One of the indicators of increasing temperature and human stressors at a local scale is a spread and recruitment of non-indigenous species (NIS) affecting economies around the world [4]. Differences in the environmental exposure may underlie the wide range of observed physiological responses highlighting the importance of quantifying the effects of climate change across systems [6]. Due to their direct and cascading effects on ecosystems and biodiversity, NIS that become invasive in aquatic environments have attracted public and scientific attention [7,8,9]. However, to better understand the role of invasive species in the function and structure of aquatic ecosystems, it is critical to understand how environmental parameters affect their physiology [10,11,12,13,14]. 

The invasive freshwater hydrozoan *Craspedacusta sowerbii* (Cnidaria, Hydrozoa, Olindiidae) is native to East Asia [15,16]. From the beginning of the 20th century, reports of *C. sowerbii* have been increasing globally [17,18,19]. *Craspedacusta sowerbii* has a metagenetic life cycle, in which asexually reproducing polyps and sexually reproducing medusae alternate [11,20]. Other, less well-studied benthic stages include frustules and podocysts. Payne (1924) and Lundberg et al. (2005) discussed that in invaded habitats, the cycle is often not completed as male and female medusae rarely co-occur [21,22]. The lack of data on biotic and abiotic factors inducing polyp colony growth and medusa production is due in part to the sporadic presence of the pelagic (i.e., medusa) stage during the summer and the difficulties in sampling the benthic (i.e., polyp) stage.

Temperature plays a key role in driving biochemical reactions and metabolic processes in organisms determining thermal tolerance limits, phenology, and their ultimate distribution [1,23,24,25]. Despite its worldwide distribution, studies on the effects of environmental parameters on *C. sowerbii* polyp and medusa stages appear to be limited [26,27,28], but likely could be the key to understanding its invasive behavior [18]. Temperature and food availability have been identified as key parameters in the asexual and sexual reproduction success of *C. sowerbii* [27,29]. A recent modeling study showed the importance of temperature on the current and future expansion of *C. sowerbii* [18]. There is little data on the thermal tolerance of both the polyp and medusa stages of *C. sowerbii*. Only two previous studies showed that the pulsation rate of medusae was positively correlated with temperature [28,30]. It is known that colonies can be composed of 1 to 7 polyps [21], but it is unknown how the polyp colony composition (i.e., number of polyps per colony and individual polyp size) may change with temperature. In the literature, a thermal optimum was identified for polyp budding between 12 and 20 °C [27], but knowledge of the effect of temperature on the fitness and structure of polyp colonies is currently missing. It appears necessary to explore the effect of temperature on polyps and medusae to predict the expansion potential of *C. sowerbii* in the near future to validate distribution models performed recently [18]. 

In this study, aiming to analyze responses of polyps and medusae to different temperatures, two data sets were used: (i) an experimental study on the polyp colony growth as a function of temperature, and (ii) a prediction of the thermal habitat suitability based on the thermal tolerance of the medusa stage. The study aims to highlight the governing influence of temperature on the polyp stage fitness and predict the thermal habitat suitability of the medusa stage.

## 2. Materials and Methods

### 2.1. Effect of Temperature on Polyp Colonies

Two laboratory experiments were conducted to investigate the effect of temperature (19 and 29 °C) on the population structure of polyps over 80 days. Polyps of *Craspedacusta sowerbii* were obtained from cultures of the Cinéaqua Aquarium (Paris, France), native to Kamo (Japan). A seed colony was maintained at 19 °C and frustules produced by the polyps were extracted with a Pasteur pipette. For each experiment and temperature treatment, 6 populations composed of 15 frustules were placed in culture tanks made of microscope glass slides (Rogo Sampaic™; length = 7.6 cm × width = 2.6 cm × height = 2.6 cm, volume = 51 mL) assembled with silicon [20]. The culture tanks (total area: 72.8 cm^2^) were placed in the culture system introduced by Marchessaux and Bejean (2020) [20]. Each population was placed in a separate culture system. As established in the literature, polyps were fed every 3 days with *Artemia salina* nauplii (1 nauplius polyp^−1^; Ocean NutritionTM, nauplius mean length: 430 µm) allowing suitable production of polyps [11,20]. To eliminate salt, nauplii were rinsed three times on a 60 µm sieve using osmosis freshwater. Nauplii were cut in half (~215 µm) using a scalpel under a stereomicroscope to facilitate ingestion by polyps.

To explore the effect of temperature on colony structure, the total number of polyps, the number of polyps per colony, as well as the number of frustules and medusa buds per polyp were counted under a stereomicroscope, usually every other day, for 80 days (data extracted from Marchessaux and Bejean, 2020). Podocysts were not quantified in this study. The percentage contribution of each colony type (1 polyp (1P), 2P, 3P, 4P, and 5P) was calculated for each observation and the mean (±standard deviation) percentage contribution of each colony type was calculated for both temperatures. To follow the normality, the data were log_10_-transformed. To test differences in the number of polyps per colony, percentage contribution of polyp type colonies, the time to produce the first polyp type, and the number of frustules and medusa buds per polyps, between temperature treatments, a one-way Analysis of Variance (ANOVA) was performed using R studio software (Boston, MA, USA; version 2021.09.0-351, package *ggpubr* [31]). If the overall ANOVA results were significant (*p* < 0.05), Bonferroni pairwise comparisons were performed to reduce the chances of obtaining false-positive results among combinations [32]. All plots were produced using R studio (package *ggplot2* [33]). 

### 2.2. Thermal Tolerance and Suitable Habitats Models of Medusae Stage

To identify the thermal tolerance of the medusa stage of *C. sowerbii*, a search of the scientific literature (tested search string: ((“Freshwater jellyfish*” OR “Craspedacusta sowerbii”) AND (“Thermal tolerance” OR “metabolism”)) was conducted using Google Scholar, Scopus, and Web of Science. Two articles were found on the thermal tolerance of the activity rates (pulsations min^−1^) of the medusa stage as a function of temperature [28,30]. The study by Dodds and Hall (1984) explored only three temperatures between 10 and 20 °C [28]. Whereas, the study of Thomas (1950) [30] was conserved in our study because the author explored the activity rates on a wide range of temperatures (28 temperatures tested between 2 and 28 °C); including the critical minimum and maximum temperatures essential for thermal performance modeling approach. The data were extracted using GetDataGraph Digitizer software (version 2.26.0.20). Thermal Performance Curve (TPC) model regression of extracted data was performed using the *rTPC* package [34] in R Studio software (Boston, MA, USA; version 2021.09.0-351). A total of 24 different non-linear least-squares thermal performance models were launched and compared. The Boatman 2017 [35] regression was identified as the “best” fitting model presenting the lowest Akaike’s Information Criterion (AIC) score (Supplementary Table S1). The non-linear Boatman 2017 regression model describes the thermal performance curves of organisms induced by enzymatic deactivation at high temperatures following the equation:

$$\mathrm{Activity}\;\mathrm{rate}=r_{\max}\times \left[\sin\times \left(\pi \times {\left(\frac{\mathrm{temperature}-\mathrm{tmin}}{t_{\max}-t_{\min}}\right)}^{a}\right)\right]{\;}^{b}$$

The maximum activity rate (r_max_), minimum (t_min_), and maximum (t_max_) temperatures, the shape parameter to adjust the skewness of the curve (a) and shape parameter to adjust the kurtosis of the curve (b) were extracted from the tolerance curve model results. Bootstrapping was used to calculate 95% prediction limits for the selected TPC model and confidence intervals around its temperature optimum. 

Based on the Boatman 2017 equation, experimental data on the activity rate were converted into probability of occurrence at various temperatures [5]. The probability of presence (Thermal Habitat Suitability, THS) was calculated in the present-day climatic conditions. Spatial occurrence probability based on the activity rate was calculated at pixel level using the mean monthly temperatures extracted from the WorldClim database (https://www.worldclim.org; resolution: 1 km^2^ [36], accessed on 1 April 2022). Low probability values (from 0 to 0.2) correspond to low activity placed in the final part of the modeled curve tails, representing the low performance of the species at those temperatures (ideally closer to the lower and higher lethal limits). 

To assess the thermal habitat suitability for *C. sowerbii* in response to future temperature increase, THS were calculated for the two future epochs (2050 and 2100) from climate models BCCCSM2-MR, the Beijing Climate Center [37]. The lower and the upper limits of the Shared Socioeconomic Pathways (SSPs; SSP126 and SSP585) were used for better prediction of future conditions. SSP126 corresponds to the optimistic case representing the sustainability route (+1 °C in 2100) and the SSP585 refers to the worst case of fossil-fueled development (+4 °C in 2100) [38]. Currently, SSPs are used by the Intergovernmental Panel on Climate Change (IPCC) in the 6th Assessment Report. Data were obtained from the Coupled Model Intercomparison Project Phase 6 (CMIP6) [39]. The monthly current and future THS maps were produced using R Studio software (version 2021.09.0-351).

## 3. Results

### 3.1. Effect of Temperature on the Polyp Colony Structure

**Figure 1:**
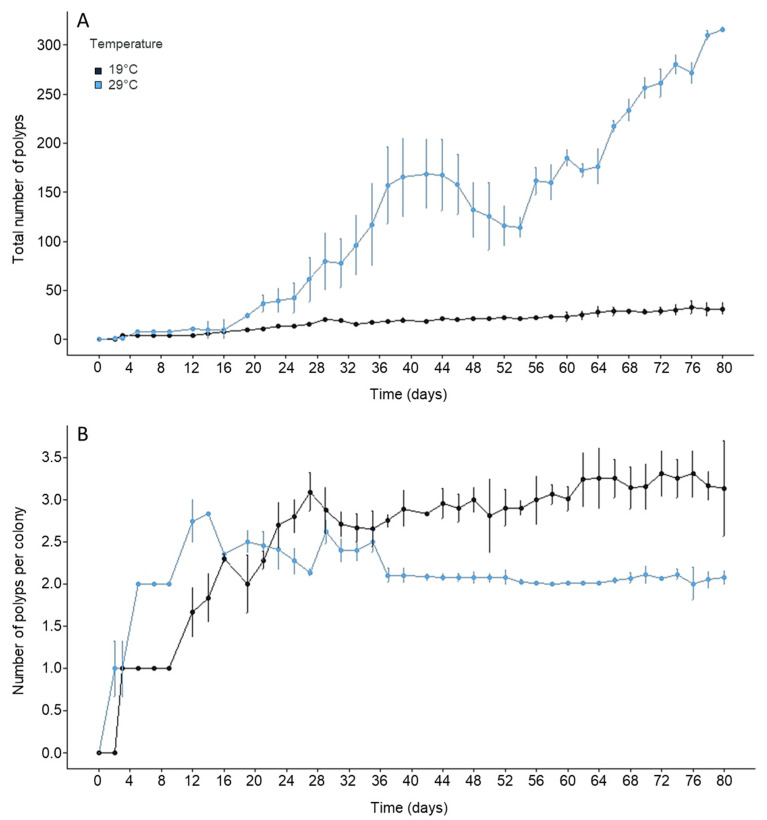
(**A**) Development of the total number of *Craspedacusta sowerbii* polyps and (**B**) mean (± standard deviation) number of polyps per colony at 19 and 29 °C, respectively.

Two (29 °C) to three (19 °C) days after inoculation, the frustules were differentiated into polyps at one end and were then considered colonies (Figure 1A). The total number of polyps at 19 °C increased from 4 (0.05 polyps/cm^2^, day 3) to 31 ± 6 polyps (0.4 ± 0.1 polyps/cm^2^, day 80) (Figure 1A). The total number was significantly higher (F = 423.71, *p* = 0.003) at 29 °C (315 ± 2 polyps; 4.3 ± 0.03 polyps/cm^2^) compared to the 19 °C treatment from day 20 by the end of the experiment. 

The number of polyps per colony increased for the first 27 days at 19 °C (maximum = 3.1 ± 0.2 polyps per colony) and for the first 13 days at 29 °C (maximum = 2.8 ± 0.1 polyps per colony) and was thereafter constant until the end of the experiment (Figure 1B). By the end of the experiment, the mean number of polyps per colony was significantly higher (F = 10.36, *p* = 0.032) at 19 °C (3.1 ± 0.6 polyps per colony) than at 29 °C (2.1 ± 0.1 polyps per colony).

**Figure 2:**
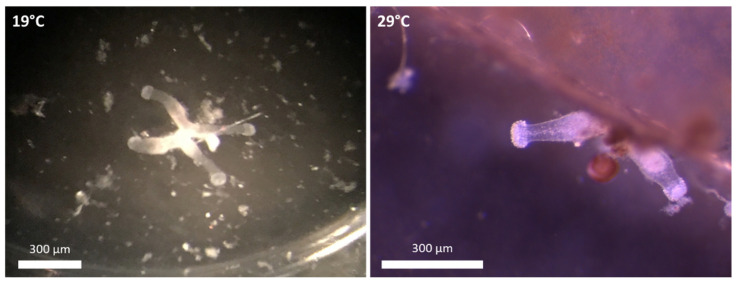
Pictures of *Craspedacusta sowerbii* polyp colony types at 19 °C (4 polyp colony), and 29 °C (2 polyp colony).

**Figure 3:**
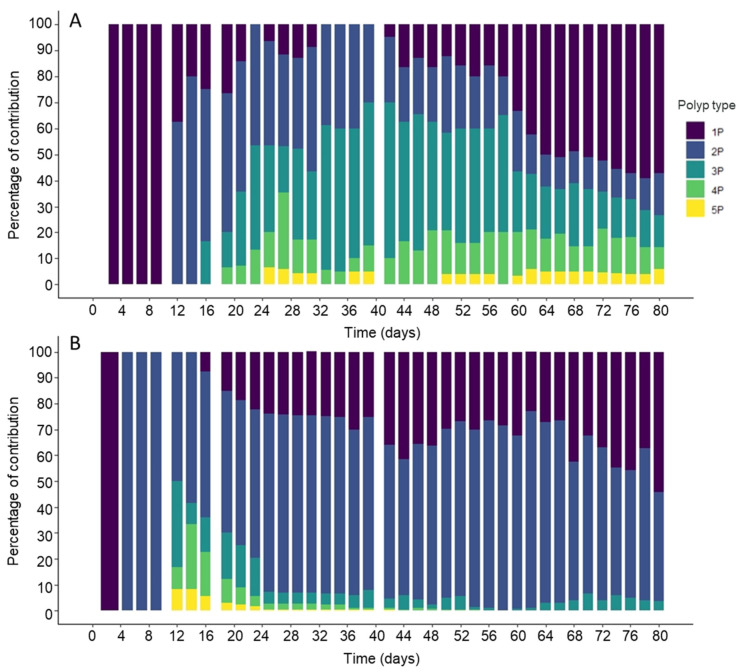
Percentage of contribution of *Craspedacusta sowerbii* polyp number per colony at (**A**) 19 °C, and (**B**) 29 °C.

The polyp colony structure differed between the two temperature treatments (Figure 2, Table 1). In the colder treatment, colonies with 1 to 5 polyps were present, with colonies of 1 to 3 polyps numerically dominating and almost equally represented (Figure 3A; Table 1): 33 ± 30% (1 polyp), 30 ± 17% (2 polyps) and 32 ± 15% (3 polyps). In the warmer treatment, colonies with 5 polyps were only present during the first 25 days and 1- to 2-polyp colonies accounted for over 80% of all colonies (30 ± 21% and 65 ± 13%, respectively). While there was no difference in the number of 1-polyp colonies at both temperatures (F = 0.32, *p* = 0.430), 2-polyp colonies were more frequent at 29°C (F = 89.22, *p* < 0.001; Table 1). The 4-polyp and 5-polyp colonies at 19 °C represented 13 ± 5% and 4 ± 2%, respectively (Figure 3A, Table 1) and their shares were significantly higher (F = 73.91, *p* < 0.001; and F = 25.06, *p* < 0.001 respectively, Table 1) than at 29 °C where less than 9% for colonies with more than 3 polyps were observed (Figure 3B, Table 1).

The time to produce the different colony types differed between both temperatures (Table 2). The first polyp appeared 1 day earlier at 29 °C (day 2) than at 19 °C, but the difference was not significant (F = 3.02, *p* = 0.158). For the higher temperature, the production of colonies with more than 1 polyp was faster than for the lower temperature treatment: it took only 5 days to observe colonies with 2 polyps and 12 days for the other forms. In contrast, at 19 °C, the time to produce 4 and 5 polyps (19 ± 2 and 25 ± 2 days, respectively) was significantly shorter (F = 7.35, *p* = 0.050; and F = 63.37, *p* = 0.001 respectively) than at 29 °C (Table 2).

The number of frustules per polyp was variable (ranging from 0.00 ± 0.00 to 0.51 ± 0.18 at 19 °C, and from 0.00 ± 0.00 to 0.28 ± 0.02 at 29 °C, Supplementary Table S2), based on the frustules present at the time of counting. The mean frustule number counted was significantly higher (F = 23.70, *p* < 0.001) at 19 °C since at high temperature, frustules escaped detection when they already transformed into polyps in the time window between counts. The much higher number of polyps over time at 29 °C (Figure 1A) might have resulted from such undetected frustules. Medusa buds were only observed at 29 °C (average: 0.012 ± 0.018 buds per polyp; Supplementary Table S2) and their number was significantly higher (F = 23.87, *p* < 0.001). The experiment presented in this study was performed on one strain of *C. sowerbii* polyps. Future studies will need to investigate how other polyp strains behave in different temperature regimes.

### 3.2. Thermal Tolerance of Medusae Stages and Thermal Habitat Suitability

**Figure 4:**
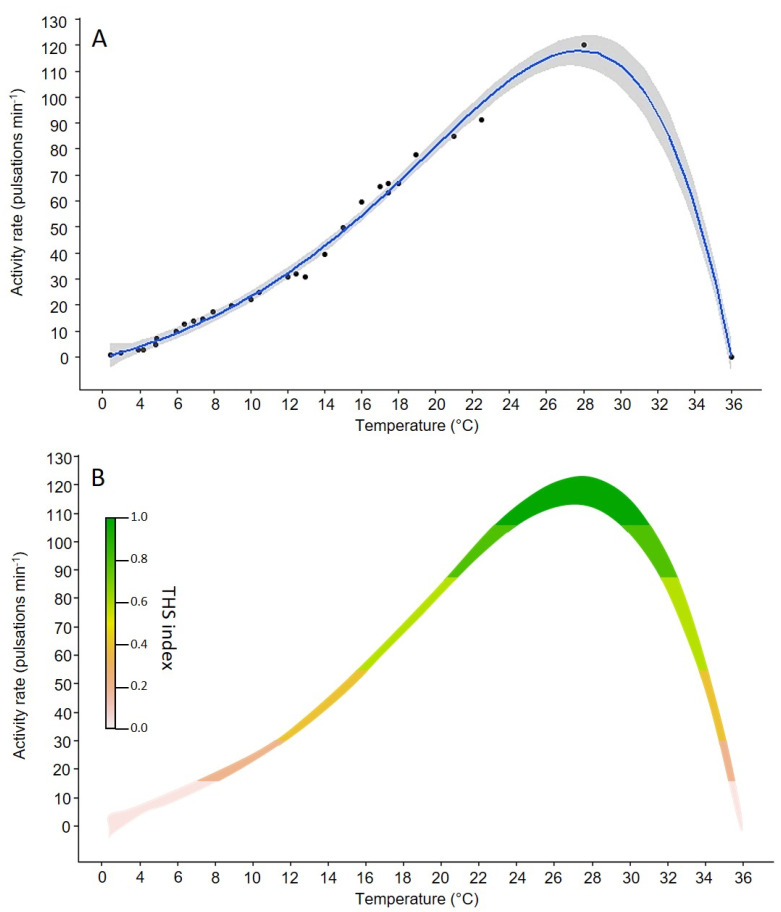
(**A**) Thermal tolerance curve (Boatman 2017 regression) of the medusa stage of *Craspedacusta sowerbii* based on the activity (pulsations min^−1^) extracted from Thomas (1950) [30]. The blue line represents the mean regression curve and the gray area indicates the 95% confidence intervals; and (**B**) Thermal habitat suitability (THS) scale within the TPC of *Craspedacusta sowerbii* ranging from low (0) to high (1) species occurrence probability (THS index). Different colors in the scale bar represent the species thermal habitat suitability.

Across the temperature range (from 2 to 28 °C) tested by Thomas (1951) [30], the medusa activity changed from 0.9 pulsations min^−1^ at 2 °C to 120 pulsations min^−1^ at 28 °C (Figure 4). At the highest temperatures (>28 °C), the activity was not measured anymore, but in the text, the author explained that the “*movement became abnormal at 31 °C to 35 °C, and ceased at 36 °C*” indicating that the species was beyond its thermal optimum of about 28 °C with a lethal temperature at 36 °C. The obtained Thermal Performance Curve (TPC) predicted a temperature optimum for the species at 28.7 °C (119 pulsations min^−1^) (Figure 4).

**Figure 5:**
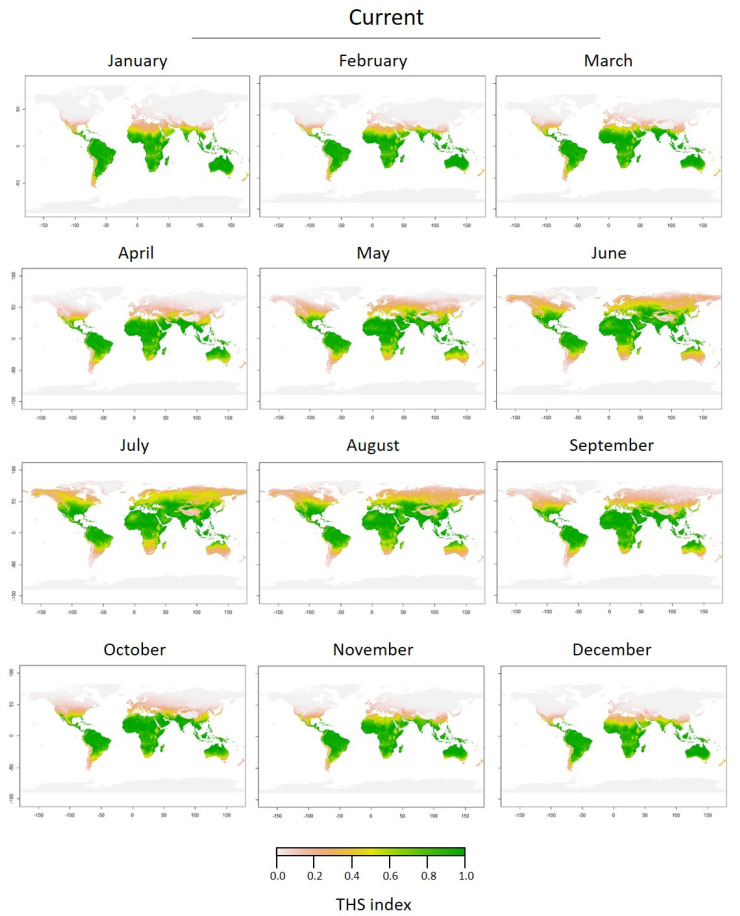
Current (2019) monthly predictions of Thermal Habitat Suitability (THS) of the medusa stage of *Craspedacusta sowerbii* based on the Thermal Performance Curve (TPC) performed on the activity rate (pulsations min^−1^, lower panel).

To create Thermal Habitat Suitability (THS) maps, activity rates were converted into the probability of presence. The different ranges of habitat suitability probability on the TPC could be identified geographically (Figure 5). Based on the thermal tolerance model (i.e., optimum thermal conditions for the species), the predicted THS maps show that the southern hemisphere offers optimal conditions (THS > 0.6) for *C. sowerbii* medusae year-round, except between August and October, where the THS decreased (~0.4) for latitudes > 50° S (Figure 5). In the northern hemisphere, the optimum period for *C. sowerbii* medusae was predicted to occur between June and September at latitudes up to 60° N. For lower northern latitudes (up to 40° N), optimal conditions (THS > 0.6) for *C. sowerbii* medusae were found year-round.

**Figure 6:**
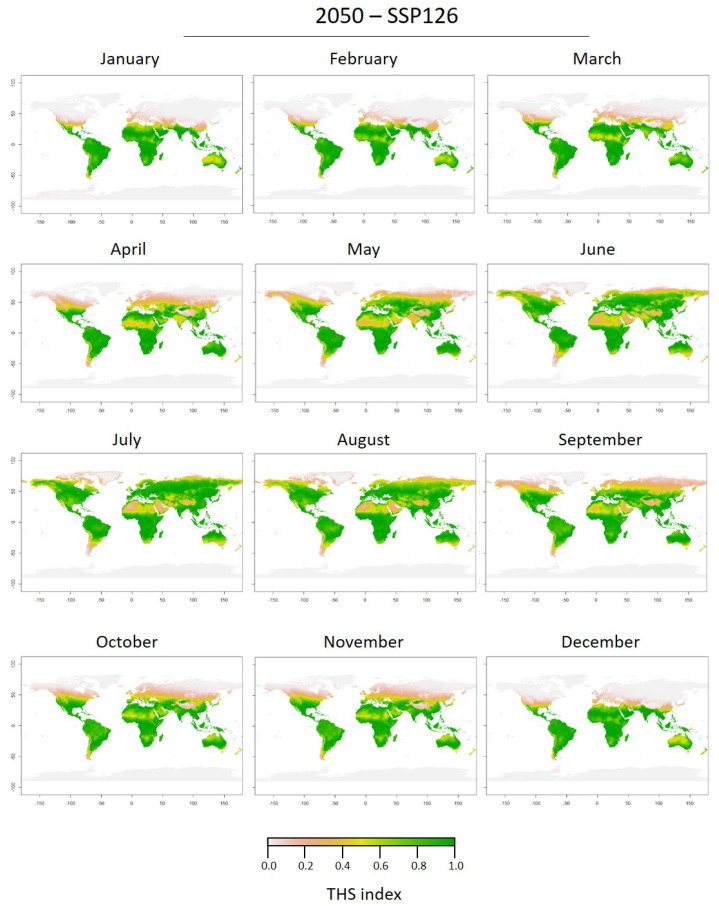
2050-SSP126 monthly predictions of Thermal Habitat Suitability (THS) of the medusa stage of *Craspedacusta sowerbii* based on the Thermal Performance Curve (TPC) performed on the activity rate (pulsations min^−1^, lower panel).

**Figure 7:**
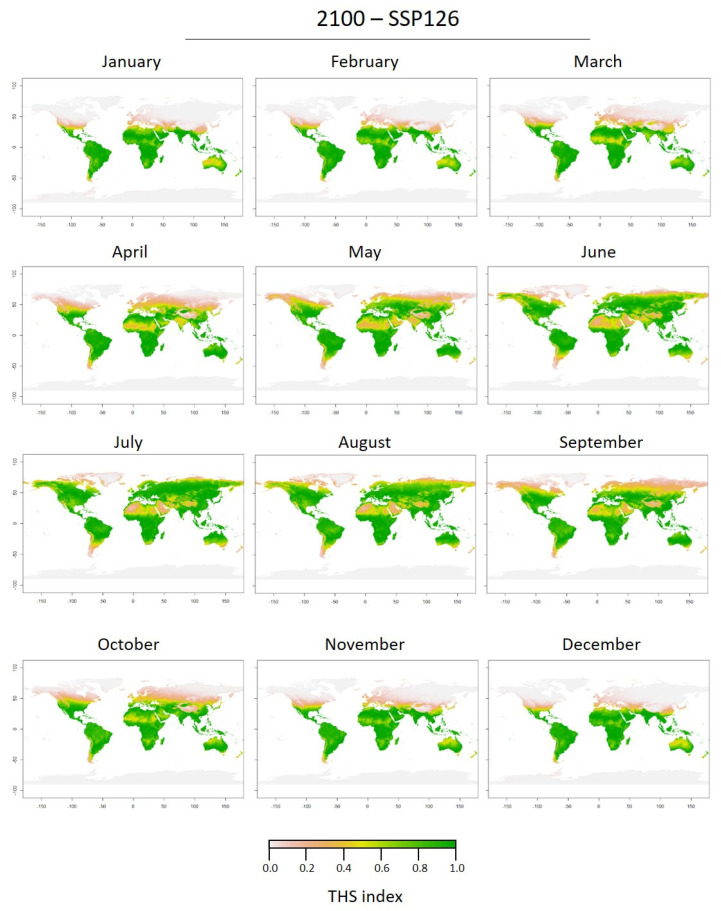
2100-SSP126 monthly predictions of Thermal Habitat Suitability (THS) of the medusa stage of *Craspedacusta sowerbii* based on the Thermal Performance Curve (TPC) performed on the activity rate (pulsations min^−1^, lower panel).

The future predicted THS maps for 2050 and 2100 as well as both scenarios (SSP126 and SSP585) showed highly similar patterns (Figure 6 and Figure 7; Supplementary Figures S1 and S2). Maps based on the high limit scenario (SSP585) were similar (Supplementary Figures S1 and S2) and the main difference between 2050 and 2100 was the increase of THS in 2100 at the global scale. The predicted distribution of *C. sowerbii* in 2050 and 2100 (SSP126) was considerably wider than at present (Figure 6 and Figure 7). For all epochs and scenarios, at latitudes close to the equator, the THS decreased in the future in contrast to the current scenario. The same seasonal patterns were observed at southern latitudes in the future with optimal THS year-round. In the northern hemisphere, THS were optimal earlier (in May) and for a longer time (until October). Also, the predicted increase in temperature in the northern hemisphere induced an increase in optimal THS for *C. sowerbii* medusae at higher latitudes (up to 60° N).

## 4. Discussion

### 4.1. Effect of Temperature on Polyp Fitness

Proliferation events of *Craspedacusta sowerbii* are irregular, unpredictable, and short-lived [40], and mentions of this cryptic species are sporadic. The presence of the polyp stage is often only suggested because they are difficult to find and sample [29]. Several factors such as temperature, pH, current velocity, and food availability influence all life cycle stages of *C. sowerbii* [41]. The temperature was suggested as the most important factor influencing polyp, frustule, and medusa budding from colonies and medusa activity [11,30,42,43,44]. Some information in the literature suggested a thermal optimum for polyp production at 20 °C; 25 °C for frustule, and 28 °C for medusa production [30,42]. Different thermal optima suggested that the preferred temperatures depend on the studied population (i.e., temperate vs. tropical) [27,41,42].

In the present study, temperature influenced the polyp population growth. Polyps of *C. sowerbii* are known to reproduce asexually in several ways, (i) budding of polyps that either stay connected to the mother polyp allowing for the formation of multi-polyp colonies or become separated, (ii) transverse division of polyps, which also forms multi-polyp colonies, (iii) frustule budding, or (iv) the budding of medusae [45,46,47,48]. Under good nutritional conditions, polyps can form new frustules (one or two at the same time) every day [47]. As the food availability in both experimental treatments was the same and without limitation, we expect no bias related to polyp nutrition. However, frustule formation is accelerated in warmer water [49] and thus impacts the numerical succession of polyp populations. Also, frustule development to the polyp stage is accelerated at high temperature. The much higher number of polyps and colonies at 29 °C can be explained by a much higher frustule production since each colony originates from a frustule. Thus, frustules were missed when the time from frustules to polyps was shorter than the 2-3 days screening window, while frustules produced at 19 °C were always counted. That the number of frustules counted was greater at 19 than at 29 °C only documents a longer developmental time at lower temperature. *Craspedacusta* polyps rarely swallow their own frustules, but in case they do, frustules are soon spluttered, indicating negligible intraspecific predation effects [50]. Frustules spent up to three weeks crawling before they transform into polyps [50]. However, most often, the frustule stage is no longer than one week [51], highlighting the need for months-long experiments to capture this dynamic. Under unfavorable conditions, e.g., a drop in temperature or low food availability, frustules do not metamorphise into polyps, but instead into podocysts, which can endure several months [47]. However, podocyst formation was difficult to quantify. To avoid any underestimation this parameter was not monitored in the present study. The final total number of polyps was ten times higher at 29 than at 19 °C as reported earlier in the literature [27]. This clearly shows that *C. sowerbii* polyps are favored numerically under increased temperature, which will have implications for food webs (discussed below). 

Colonies had a small number of polyps per colony at the beginning of the experiment (ranging from 1 to 2) and were composed of a larger number of polyps per colony after 80 days. Colonies are usually comprised of 1 to 7 polyps but can have as many as 11 polyps [45,47,48]. Despite variable polyp numbers, previous studies indicated higher numbers per colony at lower temperatures, and lower polyp per colony numbers at higher temperatures, which is confirmed by findings in the present study [11]. The numbers of polyps per colony found by the end of the experiment were consistent with those available in the literature (Table 3). 

For many aquatic organisms like *C. sowerbii*, an increase in the metabolic rate has been shown to compensate for the exposure to high temperatures [5,52]. This ability may help organisms to maintain fitness under stressed conditions [53]. In the case of *C. sowerbii* polyp colonies, reducing the number of polyps per colony at the higher temperature may allow them to reduce the metabolic demand to allocate energy for the medusa production at higher temperatures. At the same time, intra-colony competition for food is reduced to balance increased respiratory costs. As polyps of *C. sowerbii* do not have tentacles and rely on prey contact only by water movement, re-filtration of the same water parcels in colonies can be disregarded. However, intraspecific competition for food can occur also among neighboring colonies. As sessile polyps likely also rely on dissolved organic matter for nutrition, increasing the surface-to-volume ratio may be a response to physiological stress (increased temperature), thus making large colonies less desirable.

### 4.2. Effect of Temperature on the Medusa Stage

The lack of research on thermal windows of gelatinous zooplankton organisms limits our ability to predict how climate change might affect their life cycle and population dynamics [54]. It appears that the activity rate (as a proxy of metabolism) of *C. sowerbii* medusae increased exponentially with temperature to reach a maximum at 28 °C [30]. The high activity rate (119 pulsations min^−1^) at 28.7 °C suggested possible thermal stress for medusae. Beyond this temperature optimum, the activity rate decreased and ceased at 36 °C. This lethal boundary aligns well with the observations of Romanes (1880) [26], who reported a fatal temperature of 37.8 °C. Our integrated experimental and modeling effort showed that the *C. sowerbii* medusa stage should reach “peak of metabolism performance” at 28.7 °C, with a left-skewed curve characterized by a weaker performance at lower temperatures. This trait is generally representative of a “high temperature specialist” [55]. This means that beyond the species-specific thermal optimum, any further increase may result in energy reallocation, rapid reduction in growth rate, reproductive failure, and/or even mortality [56].

Based on the thermal tolerance curve of the medusa stage, current maps of optimal conditions for *C. sowerbii* cover large areas of the world, especially in the southern hemisphere and the lower latitudes (<40° N) of the northern hemisphere. Future scenarios provided larger optimal thermal areas for *C. sowerbii*, especially in the northern hemisphere, postulating a large range expansion. The generated monthly maps based on the species metabolism can be linked with the recent multiple factors correlative species distribution models that showed a poleward expansion of *C. sowerbii* by 2050 and 2100 [18]. During warmer months (35–42 °C), the medusa stage of *C. sowerbii* would be subjected to a "thermal stress state" with potentially significant effects on the reproduction and dispersal of the species, especially near the equator. Similar to other hydroids (e.g., *Ectopleura* spp., or *Pennaria disticha*) that may respond to increased temperature by autotomy and dispersal of germ cell-bearing polyps [57]. *Craspedacusta sowerbii* could also cope with and acclimate to warmer habitats by advancing the development and maturation of the free-living planktonic sexual stage and by finding new thermal habitats at higher latitudes to the north as observed in forward-looking predictions. Other environmental factors such as solar radiation, food availability, and water quality can be important when exploring multiple metabolism maps to investigate the potential of *C. sowerbii* polyps and medusae to adapt to multiple human-induced impacts [11,18,20,58,59].

## 5. Conclusions

The freshwater jellyfish *Craspedacusta sowerbii* is among the most widespread invasive species, but a general lack of information on its ecology is apparent. Based on a combined experimental and model approach, it appears that temperature profoundly influences both polyp and medusa stages. In a broader context, environmental warming will affect both life cycle stages and their food web impacts. Seasonal warming will likely allow for earlier medusa budding and prolonged continuous medusa production. Thus, polyp feeding will increase considerably with likely negative implications for other competing benthic and pelagic species [60]. Further, medusae will be longer present in the ecosystem expanding their impact on prey populations, pelagic competitors, and nutrient cycling. Indirect effects on primary and secondary production may be expected, but are less predictable. Medusae will likely dominate lake ecosystems when other species will be limited/suppressed by elevated temperatures. Thus, medusae will impact pelagic prey populations of those species that are more sensitive to environmental warming. Changing carbon fluxes and increasing medusa densities could have an effect on ecosystem dynamics where thermal generalist species will likely hide in cooler refuge areas to survive. In addition, the high density of dead medusae could promote the transfer of carbon into benthic habitats that may favor some species taking advantage of the ecological imbalance. Future research will be required to shed light on how warming will affect *C. sowerbii* food web impacts.

The present study answered some questions related to polyp and medusa physiology and adaptation to environmental warming. However, multi-factor experiments and modeling approaches are needed to more realistically predict how both life cycle stages will be affected. Even if the medusa stage is the easier stage to sample, it appears that there is a general lack of knowledge on its metabolism. Response studies to environmental change of frustules and podocysts are almost entirely missing. In the context of climate change, the polyp stage could be the key to understanding the response and adaptations of this species and calls for more research efforts.

## Figures and Tables

**Table 1:** Mean (±standard deviation) percentage contribution of the number of *Craspedacusta sowerbii* polyps per colony at 19 and 29 °C, respectively. *: significant difference (*p* < 0.05).

Colony Types	19 °C	29 °C	F Value	*p* Value
1 polyp	33 ± 30	30 ± 21	0.32	0.430
2 polyps	30 ± 17	65 ± 13	89.22	<0.001 *
3 polyps	32 ± 15	6 ± 6	93.57	<0.001 *
4 polyps	13 ± 5	3 ± 5	73.91	<0.001 *
5 polyps	4 ± 2	1 ± 2	25.06	<0.001 *

**Table 2:** Mean (±standard deviation) time (days) to produce the first *Craspedacusta sowerbii* polyp colony type at 19 °C and 29 °C, respectively. *: significant difference (*p* < 0.05).

Colony Types	19 °C	29 °C	F Value	*p* Value
1 polyp	3 ± 1	2 ± 0	3.02	0.158
2 polyps	12 ± 2	5 ± 2	29.40	0.006 *
3 polyps	16 ± 5	12 ± 5	0.96	0.383
4 polyps	19 ± 2	12 ± 4	7.35	0.050 *
5 polyps	25 ± 2	12 ± 2	63.37	0.001 *

**Table 3:** Review on multi-polyp colony development at various temperatures.

Temperature (°C)	Mean Time (Days) to First Polyp Formation	Mean Number of Polyps Per Colony	Reference
12	-	5.9	[27]
19	3.1 ± 0.6	3.1 ± 0.2	This study
20	-	2 - 4	[27]
24	5.1 ± 0.5	2.2 ± 0.1	[11]
25	-	1.8 to 3.8	[27]
26	-	2 to 6	[11]
28	-	3.8	[27]
29	2.1 ± 0.1	2.2 ± 0.2	This study

## Data Availability

The data presented in this study are available on request from the corresponding author.

## Supplementary Materials

The following are available online at https://www.mdpi.com/article/10.3390/biology11081100/s1, Table S1: Parameters used for the Thermal Performance Curve (TPC) model selection; Table S2: Number of frustule and medusa buds per polyp of *Craspedacusta sowerbii* at 19 and 29 °C over time (means ± SD); Figure S1: 2050-SSP585 monthly predictions of thermal habitat suitability of the medusa stage of *Craspedacusta* sowerbii; Figure S2: 2100-SSP585 monthly predictions of thermal habitat suitability of the medusae stage of *Craspedacusta sowerbii*.
