# Supplementary material for: Increasing Temperature Facilitates Polyp Spreading and Medusa Appearance of the Invasive Hydrozoan Craspedacusta sowerbii
Source: Biology (Basel). 2022 Jul 23;11(8):1100. doi: 10.3390/biology11081100 (PMC9331908; doi:10.3390/biology11081100)
Supplement: Supplementary file 1 [file biology-11-01100-s001.zip › biology-1731742-supplementary.pdf]

biology

Supplementary Materials

Increasing Temperature Facilitates Polyp Spreading and Medusa Appearance of the Invasive Hydrozoan *Craspedacusta sowerbii*

Guillaume Marchessaux, Florian Lüskow, Mickaël Bejean and Evgeny A. Pakhomov

Table S1. Parameters used for the Thermal Performance Curve (TPC) model selection. Sigma: residual standard deviation; AIC: Akaike's Information Criterion; AICc: corrected AIC for small sample sizes; df.residual: residual degrees-of-freedom.

model_name	sigma	AIC	AICc	df.residual
**Boatman**	**2.84**	**150.11**	**155.45**	**23**
Sharpeschoolfull	2.91	150.85	154.67	24
Beta	2.97	151.99	155.80	24
Thomas2	2.98	152.23	156.04	24
Ratkowsky	3.22	155.77	158.38	25
Thomas1	3.78	165.10	167.71	25
Oneill	4.38	173.63	176.24	25
Weibull	5.31	184.79	187.40	25
Rezende	6.20	193.82	196.43	25
Pawar	6.31	194.82	197.43	25
Sharpeschoolhigh	6.31	194.82	197.43	25
Spain	6.96	200.54	203.15	25
Briere2	9.26	217.08	219.69	25
Gaussian	9.50	217.72	219.39	26
Modifiedgaussian	9.53	218.76	221.37	25
Flinn	10.83	225.31	226.97	26
Hinshelwood	12.55	234.70	237.31	25
Quadratic	16.73	250.52	252.19	26
Sharpeschoollow	18.65	257.68	260.29	25
Johnson_lewin	21.39	265.66	268.27	25
Joehnk	21.35	266.36	270.18	24
Lactin2	23.90	272.07	274.68	25
Kamykowski	37.88	299.61	303.43	24

Table S2. Number of frustule and medusa buds per polyp of *Craspedacusta sowerbii* at 19 and 29°C over time (means ± SD).

Time (days)	Number of frustules per polyp		Number of medusae buds per polyp	
	19°C	29°C	19°C	29°C
0	0.00 ± 0.00	0.00 ± 0.00	0.000 ± 0.000	0.000 ± 0.000
2	0.00 ± 0.00	0.00 ± 0.00	0.000 ± 0.000	0.000 ± 0.000
3	0.00 ± 0.00	0.00 ± 0.00	0.000 ± 0.000	0.000 ± 0.000
5	0.00 ± 0.00	0.00 ± 0.00	0.000 ± 0.000	0.000 ± 0.000
7	0.00 ± 0.00	0.00 ± 0.00	0.000 ± 0.000	0.000 ± 0.000
9	0.00 ± 0.00	0.00 ± 0.00	0.000 ± 0.000	0.000 ± 0.000
12	0.00 ± 0.00	0.07 ± 0.12	0.000 ± 0.000	0.000 ± 0.000
14	0.00 ± 0.00	0.00 ± 0.00	0.000 ± 0.000	0.000 ± 0.000
16	0.10 ± 0.17	0.00 ± 0.00	0.000 ± 0.000	0.000 ± 0.000
19	0.00 ± 0.00	0.19 ± 0.05	0.000 ± 0.000	0.000 ± 0.000
21	0.11 ± 0.19	0.20 ± 0.03	0.000 ± 0.000	0.000 ± 0.000
23	0.13 ± 0.15	0.20 ± 0.03	0.000 ± 0.000	0.000 ± 0.000
25	0.23 ± 0.14	0.16 ± 0.08	0.000 ± 0.000	0.000 ± 0.000
27	0.21 ± 0.12	0.16 ± 0.07	0.000 ± 0.000	0.000 ± 0.000
29	0.16 ± 0.09	0.15 ± 0.08	0.000 ± 0.000	0.000 ± 0.000
31	0.25 ± 0.11	0.15 ± 0.01	0.000 ± 0.000	0.000 ± 0.000
33	0.17 ± 0.04	0.11 ± 0.05	0.000 ± 0.000	0.000 ± 0.000
35	0.18 ± 0.11	0.14 ± 0.08	0.000 ± 0.000	0.000 ± 0.000
37	0.21 ± 0.10	0.08 ± 0.04	0.000 ± 0.000	0.000 ± 0.000
39	0.27 ± 0.18	0.01 ± 0.01	0.000 ± 0.000	0.000 ± 0.000
42	0.21 ± 0.16	0.02 ± 0.03	0.000 ± 0.000	0.000 ± 0.000
44	0.29 ± 0.03	0.02 ± 0.01	0.000 ± 0.000	0.000 ± 0.000
46	0.16 ± 0.06	0.02 ± 0.02	0.000 ± 0.000	0.000 ± 0.000
48	0.42 ± 0.13	0.01 ± 0.02	0.000 ± 0.000	0.006 ± 0.010
50	0.51 ± 0.18	0.02 ± 0.01	0.000 ± 0.000	0.023 ± 0.020
52	0.49 ± 0.09	0.04 ± 0.02	0.000 ± 0.000	0.051 ± 0.041
54	0.47 ± 0.03	0.01 ± 0.01	0.000 ± 0.000	0.041 ± 0.031
56	0.40 ± 0.11	0.02 ± 0.02	0.000 ± 0.000	0.037 ± 0.014
58	0.41 ± 0.07	0.03 ± 0.02	0.000 ± 0.000	0.005 ± 0.009
60	0.23 ± 0.13	0.00 ± 0.01	0.000 ± 0.000	0.019 ± 0.007
62	0.19 ± 0.04	0.00 ± 0.00	0.000 ± 0.000	0.000 ± 0.000
64	0.25 ± 0.09	0.01 ± 0.01	0.000 ± 0.000	0.028 ± 0.025
66	0.16 ± 0.04	0.06 ± 0.05	0.000 ± 0.000	0.042 ± 0.018
68	0.15 ± 0.02	0.01 ± 0.02	0.000 ± 0.000	0.051 ± 0.025
70	0.16 ± 0.10	0.02 ± 0.02	0.000 ± 0.000	0.028 ± 0.009
72	0.18 ± 0.06	0.02 ± 0.02	0.000 ± 0.000	0.059 ± 0.052
74	0.14 ± 0.01	0.03 ± 0.06	0.000 ± 0.000	0.014 ± 0.024
76	0.18 ± 0.06	0.01 ± 0.02	0.000 ± 0.000	0.023 ± 0.040
78	0.15 ± 0.06	0.02 ± 0.03	0.000 ± 0.000	0.019 ± 0.032
80	0.09 ± 0.04	0.28 ± 0.02	0.000 ± 0.000	0.030 ± 0.026
**Mean (± SD)**	**0.18 ± 0.14**	**0.06 ± 0.07**	**0.000 ± 0.000**	**0.012 ± 0.018**

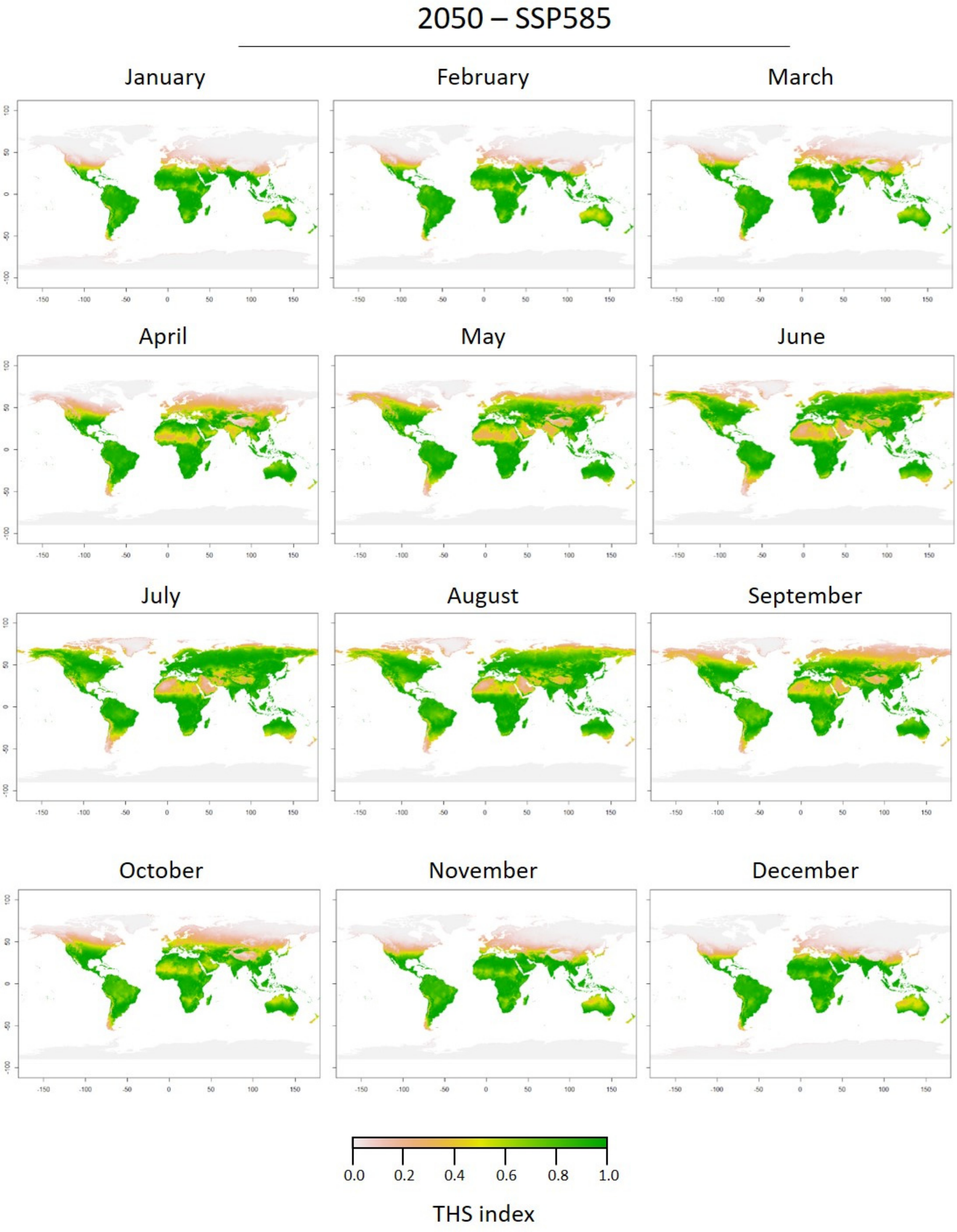

Figure S1. 2050-SSP585 monthly predictions of thermal habitat suitability of the medusa stage of *Craspedacusta sowerbii* based on the Thermal Performance Curve (TPC) performed on the activity rate (pulsations min^{-1}, lower panel).

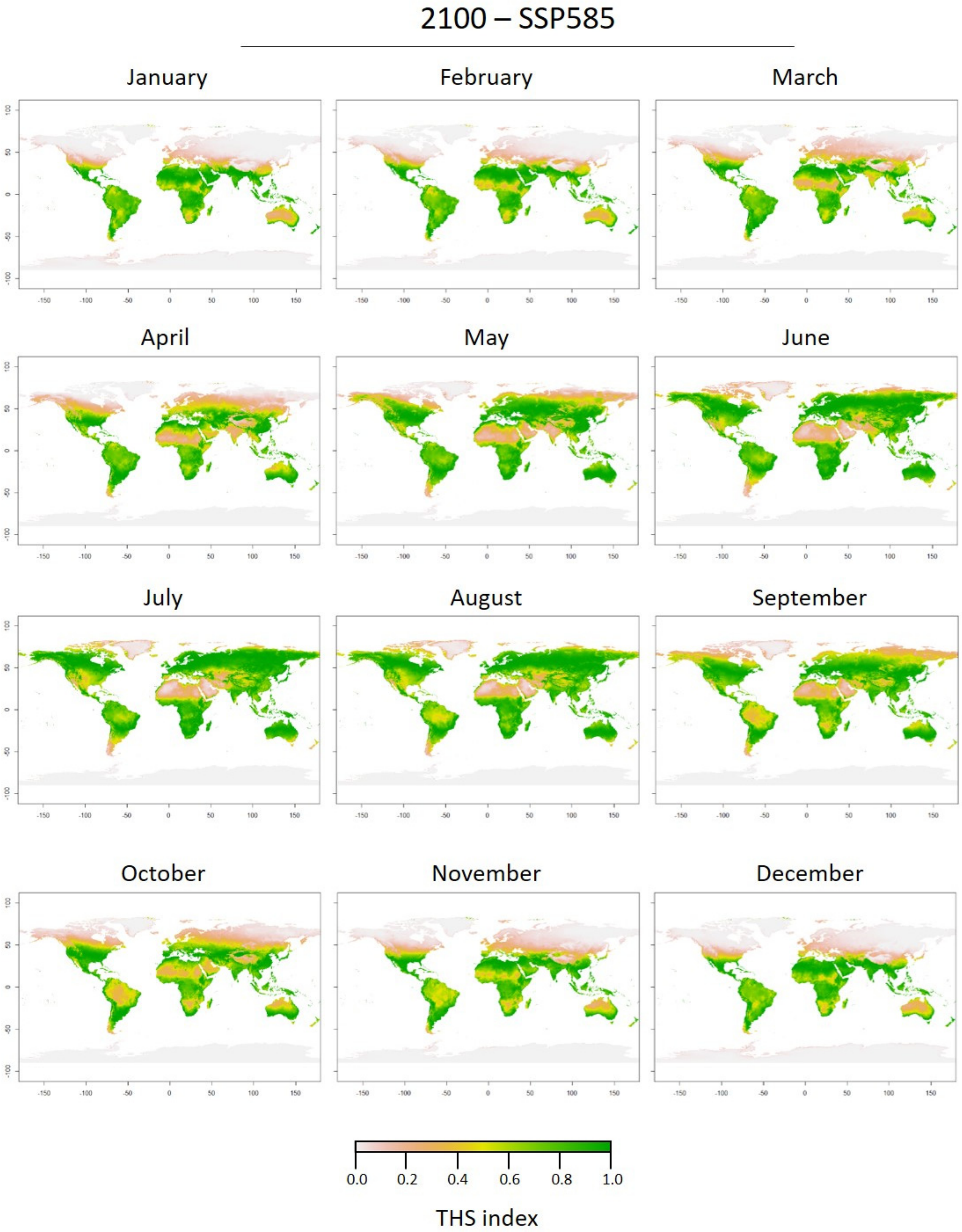

Figure S2. 2100-SSP585 monthly predictions of thermal habitat suitability of the medusa stage of *Craspedacusta sowerbii* based on the Thermal Performance Curve (TPC) performed on the activity rate (pulsations min^{-1}, lower panel).
